# Triple Therapy with Scopolamine, Ondansetron, and Dexamethasone for Prevention of Postoperative Nausea and Vomiting in Moderate to High-Risk Patients Undergoing Craniotomy Under General Anesthesia: A Pilot Study

**DOI:** 10.3389/fmed.2015.00040

**Published:** 2015-06-15

**Authors:** Sergio D. Bergese, Maria A. Antor, Alberto A. Uribe, Vedat Yildiz, Joseph Werner

**Affiliations:** ^1^Department of Anesthesiology, The Ohio State University Wexner Medical Center, Columbus, OH, USA; ^2^Department of Neurological Surgery, The Ohio State University Wexner Medical Center, Columbus, OH, USA; ^3^Department of Anesthesiology, Jackson Memorial Hospital, University of Miami, Miami, FL, USA; ^4^Center for Biostatistics, The Ohio State University, Columbus, OH, USA

**Keywords:** nausea, vomiting, scopolamine, postoperative care, ondansetron

## Abstract

**Introduction:**

Postoperative nausea and vomiting (PONV) is one of the most common complaints from patients and clinicians after a surgical procedure. According to the current Society of Ambulatory Anesthesia Consensus Guidelines, the general incidence of vomiting and nausea is around 30 and 50%, respectively; and up to 80% in high-risk patients. In previous studies, the reported incidence of PONV at 24 h after craniotomy was 43–70%. The transdermal scopolamine (TDS) delivery system contains a 1.5-mg drug reservoir, which is designed to deliver a continuous slow release of scopolamine through intact skin during the first 72 h of patch application. Therefore, we designed this single arm, non-randomized, pilot study to assess the efficacy and safety of triple therapy with scopolamine, ondansetron, and dexamethasone to prevent PONV.

**Materials and methods:**

In the preoperative area, subjects received an active TDS 1.5 mg that was applied to a hairless patch of skin in the mastoid area approximately 2 h prior to the operation. Immediately after anesthesia induction, all patients received a single 4 mg dose of ondansetron IV and a single 10 mg dose of dexamethasone IV. Patients who experienced nausea and/or vomiting received ondansetron 4 mg IV as the initial rescue medication. Postoperative nausea and vomiting assessments were performed for up to 120 h after surgery.

**Results:**

A total of 36 subjects were analyzed. The overall incidence of PONV during the first 24 h after neurological surgery was 33% (*n* = 12). The incidence of nausea and emesis during the first 24 h after surgery was recorded as 33% (*n* = 12) and 16% (*n* = 6), respectively.

**Conclusion:**

Our data showed that this triple therapy regimen may be an efficient alternative regimen for PONV prophylaxis in patients undergoing neurological surgery with general anesthesia. Further studies using regimens affecting different receptor pathways should be performed to better prove the efficacy and safety in the prevention or delay of PONV.

## Introduction

Postoperative nausea and vomiting (PONV) is one of the most common concerns from subjects and clinicians after a surgical procedure (1). The literature defines PONV as the presence of nausea and vomiting during the first 24 hours after a surgical procedure. PONV can be classified as either early PONV (0–2 hours (h)) or delayed PONV (2–24 h) (1). Episodes that occur after the patient has left the institution are known as post-discharge nausea and vomiting (PDNV) (1, 2). According to the 2014 consensus guidelines for the management of PONV from the Society of Ambulatory Anesthesia (SAMBA), the general incidence of vomiting and nausea is around 30 and 50%, respectively; and up to 80% in high-risk patients (three and more risk factors) (1). The increase in central venous pressure (CVP) that results from the act of retching can lead to potentially severe complications in certain surgical populations. For instance, in craniotomy patients, this increase in CVP could presumably lead to postoperative intracranial hemorrhage or worsening of intracranial pressure (3). In previous studies, the reported incidence of PONV at 24 h after craniotomy was 43–70% (4–7).

Ondansetron is a serotonin (5HT3) receptor antagonist most used in PONV trials and is considered the “gold standard” antiemetic due to its efficacy and low cost (1, 6, 9, 10, 14–18). This drug’s short half-life of around 4 h and its lack of sedative effect make it an excellent option in the neurosurgical field (1, 4, 10). The corticosteroid dexamethasone is another medication widely used for PONV prophylaxis; its intravenous (IV) administration after anesthesia induction is recommended, and it has similar efficacy for PONV prophylaxis as ondansetron (1, 4). Scopolamine is considered a non-polar, tertiary amino compound, and muscarinic acetylcholine antagonist. As part of its pharmacodynamic properties, it has an adequate absorption in the gastrointestinal tract and excellent blood–brain barrier penetration. Scopolamine has also been reported to be effective in the prevention of PONV (1, 2, 5, 10, 15, 17, 19–22). The literature showed that IV or intramuscular scopolamine has a short half-life in plasma and may lead to dose-dependent side effects such as excessive drowsiness, agitation, hallucinations, dizziness, dry mouth, and lethargy. Conversely, transdermal scopolamine (TDS) releases the drug over a period of 72 h; for this reason, the risks of plasma concentration-related side effects may be lower due to the slower rate of transdermal absorption (10). TDS contains a 1.5-mg drug reservoir, which is intended to supply a continuous slow release of scopolamine through intact skin during the first 72 h of patch application (23). The U.S. Food and Drug Administration (FDA) approved the TDS in 1979 as a method of motion sickness prophylaxis; then, in 2001, it was approved for PONV prophylaxis (2). PDNV usually remains as unreported events due to early discharge despite the fact that it is usually documented by many patients as the most undesirable postoperative event (2, 24). TDS is designed to deliver a constant rate of approximately 1.0 mg of scopolamine into the systemic circulation over 72 h and thus may play an important role to prevent PDNV since it is designed to deliver scopolamine for 72 h. Multiple studies using TDS for prophylaxis did not assess PONV 24 h after surgery, thus data supporting the antiemetic effects for delayed PONV are lacking (5, 19, 20, 22, 25). Considering the beneficial effects of TDS, its favorable pharmacokinetic and pharmacodynamic profile, and the importance of PONV prophylaxis, TDS may turn into a strong alternative to be one of the first or second line therapeutic agents for PONV (2). The efficacy of TDS in PONV prophylaxis is well-established in different studies (1, 5, 9, 15, 19–22, 26). However, the use of TDS in the neurosurgical field has not been studied before, probably due to its controversial sedative side effect and the rare possibility of untoward physical exam findings from this anticholinergic medication (e.g., dilated pupils) (2). Therefore, we designed this single arm, non-randomized, pilot study to assess the efficacy and safety of triple therapy with scopolamine, ondansetron, and dexamethasone to prevent PONV. We hypothesized that prophylactic triple therapy with scopolamine, ondansetron, and dexamethasone was an effective and safe treatment for the prevention of PONV in patients with moderate to high risk of PONV who underwent neurological surgery under general anesthesia (27).

## Materials and Methods

After obtaining institutional review board (Office of Responsible Research Practices) approval for the research protocol, a total of 44 subjects provided their written informed consent before any study-specific procedures were done at The Ohio State University Wexner Medical Center. Study inclusion criteria consisted of neurosurgical subjects scheduled to undergo elective craniotomy (opening of the cranium and Dura mater) requiring at least 1 h of general anesthesia with an expected 24 h hospitalization. These subjects had moderate to severe risk for PONV as assessed by having two or more risk factors on the simplified Apfel score. Exclusion criteria consisted of prisoner status, medical history of alcohol or drug abuse, history of allergic reaction or intolerance to any study medications, pregnant or breastfeeding female subjects, history of nausea and/or vomiting within 24 h prior craniotomy, history of treatment with antiemetic medication for nausea or vomiting within 24 h of their procedure, and history of chemotherapy treatment within 4 weeks prior to surgery. Subjects who had received any medication with antiemetic properties were also excluded from participating in the study.

Prior to surgery, vitals and study safety procedures including electrocardiogram (ECG) and a urine or serum pregnancy test were performed. In the preoperative area, subjects received an active TDS 1.5 mg that was applied to a hairless patch of skin in the mastoid area approximately 2 h prior to the operation. Immediately after anesthesia induction, all subjects received a single 4 mg dose of ondansetron IV and a single 10 mg dose of dexamethasone IV.

The standardized anesthesia regimen also consisted of pre-medication of midazolam 1–2 mg IV immediately before transferring the subjects to the operating room. Anesthesia was induced with propofol 1–2 mg/kg IV and fentanyl 0.75–1.5 μg/kg IV. Tracheal intubation was performed after the administration of rocuronium 0.6–1.2 mg/kg^−1^ IV.

General anesthesia was maintained with volatile anesthetics (sevoflurane, desflurane, or isoflurane) and its titration concentration was guided on clinical judgment. Analgesia during anesthesia maintenance was provided with fentanyl boluses of 0.5–2.0 μg/kg^−1^ IV. At the end of the procedure, neostigmine and glycopyrrolate were used to reverse residual neuromuscular block.

After surgery, subjects were transferred to the surgical intensive care unit (SICU) or post anesthesia care unit (PACU); subjects that experienced nausea and/or vomiting received ondansetron 4 mg IV as the initial rescue medication for PONV. Choice of subsequent rescue antiemetic was left to the anesthesiologist’s discretion.

This pilot study explored the effects of TDS triple therapy on PONV 24 h postoperative as a primary endpoint. As such, TDS was removed by the study investigators 24 h after the end of surgery (defined as discontinuation of the inhaled anesthetic). If the patch became dislodged from the skin during the first 24 h after surgery, study investigators reapplied in the same site and covered with a bandage. Anesthesia and surgical procedure start and end time, admission and discharge time from the PACU, SICU, general care floor, total length of stay, and preoperative Apfel score were recorded. As a secondary outcome, the influence of this triple therapy administration on delayed PONV was assessed every 24 h for 5 days via direct interview and/or medical records review. Intraoperative medication and opioid daily consumption from PACU arrival time through a 5-day follow up were recorded.

First episode of nausea, vomiting, and rescue medication were recorded. Nausea was assessed by asking subjects to rate their nausea on a 0–10 point scale, with 0 being no nausea at all and 10 being severe nausea. Vomiting was assessed by asking subjects to rate their vomiting on a 0–3 point scale, with 0 being no vomiting, 1 being mild vomiting (1–2 episode in 12 h, small amount of emesis), 2 being moderate vomiting (3–5 episodes in 12 h, breakthrough vomiting), and 3 being severe vomiting (6–7 episodes in 12 h, intractable, incessant, projectile).

For subjects discharged before the end of a 5-day time period, telephone contact was performed every 24 h to assess nausea and/or vomiting, rescue medication, opioid consumption, as well as adverse events and serious adverse events. Following the first 24 h after administration of the prophylactic triple therapy, an ECG was performed as part of safety assessments.

## Results

**Figure 1:**
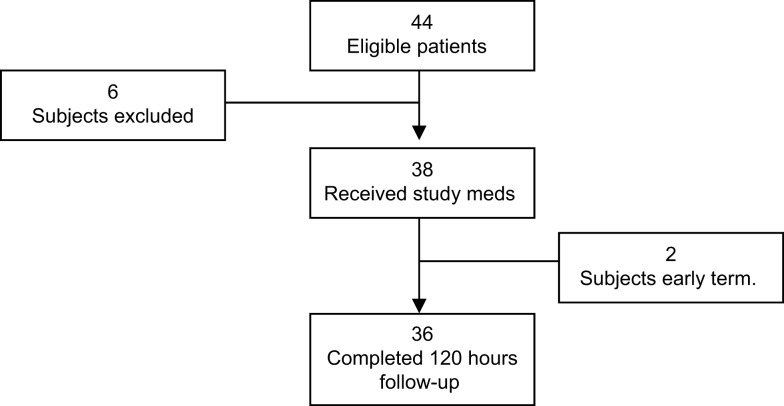
**Patient screening flowchart**.

A total of 44 subjects were enrolled in the study, 6 were considered screen failures, and 2 were early terminations. Thus, the data from 36 subjects were analyzed. The primary reason for screen failure was failure to meet all of the inclusion/exclusion criteria (*n* = 6). One subject was excluded from the study due to the surgeon’s decision to remove the TDS before surgery and another due to change in the surgical procedure from craniotomy to craniectomy (Figure 1).

Subjects were further analyzed based on whether or not they experienced any nausea during the first 120 h after the surgery. The subjects demographic’s, Apfel risk factors for PONV, duration of anesthesia, duration of PACU stay, duration of SICU stay, duration of total hospital stay, and postoperative opioid consumption are listed in Table 1. No adverse events related to the study medications were observed.

**Table 1:** **Patient demographics and surgical variables**.

Demographics and surgical variables	Scopolamine group (*n* = 36)
Age, mean (SD), years	48 (14.6)
Weight, mean (SD), kg	82 (20.4)
Height, mean (SD), cm	169 (9.6)
BMI, mean (SD)	29 (8.2)
ASA I/II/III	1/11/24
Race-White, *n* (%)	29 (80)
Sex (female) *n* (%)	22 (61)
Non-smoking status, *n* (%)	33 (92)
History of PONV and/or motion of sickness *n* (%)	12 (33)
Postoperative opioids, *n* (%)	36 (100)
Apfel risk factors, *n* (%)	
2	13 (36.1)
3	15 (42.0)
4	8 (22.0)
Duration of anesthesia, mean (SD), hours	5 (2.8)
Duration of PACU stay, mean (SD), hours	2 (1.0)
Duration of SICU stay, mean (SD), days	2 (2.3)
Duration of total hospital stay, mean (SD), days	3 (3)
Postoperative opioid consumption 0–24, media (IQR) oral morphine, mg	66 (41–154)
Postoperative opioid consumption 0–48, media (IQR) oral morphine, mg	123 (45–241)
Postoperative opioid consumption 0–120, media (IQR) oral morphine, mg	228 (48–416)

The overall incidence of PONV during the first 24 h after craniotomy was 33% (*n* = 12). The incidence of nausea and emesis during the first 24 h after surgery was recorded as 33% (*n* = 12) and 16% (*n* = 6), respectively. The percentage of subjects with freedom from emesis episodes over 0–24 h postoperatively was 84% (*n* = 30, 83% CI; 67–93%). Of those that experienced nausea during the first 24 h after neurological surgery, the median severity was rated as 8.5 (5.5–10). The median severity of vomiting was 2 (1–2), corresponding to moderate severity. Rescue medication during the first 24 h was used in 11 subjects (30%). The complete response and control rate during the first 24 h as 69% (*n* = 25, 95% CI: 53–84%) (Table 2).

**Table 2:** **Postoperative nausea and vomiting outcome variables (*n* = 36)**.

Outcome variables	0–2 h		0–24 h		24–48 h		24–72 h		24–96 h		24–120 h	
	*N*	%	*N*	%	*N*	%	*N*	%	*N*	%	*N*	%
Freedom from emesis	35	98	30	84	33	92	32	89	31	86	30	84

Vomiting	1	2	6	16	3	8	4	11	5	14	6	16

Number of vomiting episodes (IQR)	2 (2, 2)		1.5 (1, 3)		1 (1, 4)		1.5 (1, 3)		1 (1, 4)		1 (1, 4)	

Vomiting severity (IQR)	2 (2, 2)		2 (1, 2)		3 (2, 3)		2.5 (2, 3)		2 (2, 3)		2.5 (2, 3)	

Nausea	4	11	12	33	8	22	12	33	14	38	14	38

Number of nausea episodes (IQR)	2 (1.5, 2.5)		2 (1.5, 3)		2 (1, 4.5)		1 (1, 2)		3.5 (1, 6)		3.5 (1, 8)	

Worst nausea score (IQR)	6 (5.5, 8)		8.5 (5.5, 10)		7.5 (5, 10)		7 (5, 9.5)		7 (5, 9)		7 (5, 9)	

Complete control	32	89	25	69	32	89	30	83	28	78	27	75

Complete response	32	89	25	69	32	89	30	83	28	78	27	75

Number of rescue therapy	4	11	11	30	2	5	3	8	4	9	4	9

*Complete control = no emesis + no rescue medication + no nausea > 4 (0–10 scale)*.

*Complete response = no emesis + no rescue medication*.

The mean time to first emetic episode, first rescue, and first significant nausea was 26.7 (±30.2), 19.6 (±24.1), 23.6 (±27.1) hours, respectively (Table 3).

**Table 3:** **Intent to treat population**.

Time to treatment failure, mean (SD)	Time hours Scopolamine (*n* = 36)
Time to first emetic episode	26.7 (30.2)
Time to first rescue	19.6 (24.1)
Time to first significant nausea	23.6 (27.1)

*95% Confidence interval for complete response (0–24 h): [0.53, 0.84]*.

The cumulative overall incidence of PONV during the delayed period (24–120 h) was 38% (*n* = 14). The incidence of nausea and emesis during 24–120 h after surgery was recorded as 38% (*n* = 14) and 16% (*n* = 6), respectively. The percentage of subjects with freedom from emesis episodes over 24–120 h postoperative was 84% (*n* = 30, 83% CI; 67–93%). Of those that experienced nausea during 24–120 h after neurological surgery, the median severity was rated as 7 (5, 9). The median severity of vomiting was 1 (1, 4), corresponding to mild severity. Rescue medication during 24–120 h was used in 4 subjects (9%). The complete response and complete control rate during 24–120 h was 75% (*n* = 27, 95% CI: 74–96%).

The median number of vomiting episodes from 0 to 120 h postoperative ranged between 1 and 1.5 (Table 2).

## Discussion

The results of this study demonstrated that the combined use of TDS, ondansetron, and dexamethasone was an effective therapy to prevent nausea and vomiting in subjects undergoing craniotomy using general anesthesia. This prospective pilot study analyzed the data of 36 subjects that underwent craniotomy; postoperative nausea and vomiting assessments were performed for up to 120 h after surgery.

There is not a single risk factor for PONV that is sensitive or specific enough to be used alone to assess the risk of PONV. However, one can combine multiple independent predictors using the simplified risk score model from Apfel et al. to help stratify a patient’s risk for PONV (8). The risk factors from the simplified risk score model are female gender, smoking status, postoperative opioid consumption, and a history of PONV/PDNV or motion sickness (8). Each factor has a value of 1, which gives a range of 0–4 when added together, 0 being low-risk and 4 signaling high-risk patients (1, 8–10). The SAMBA Consensus Guidelines for the Management of PONV recommend assessing patient’s risk factors and providing treatment to prevent PONV. In addition, SAMBA recommends prophylactic combined therapy in subjects with moderate and high risk for PONV (1, 11). Considering the potentially catastrophic effects of vomiting on intracranial pressure and postoperative hemorrhage for post-craniotomy patients, a long-lasting antiemetic profile might be favorable for postoperative patient care and may diminish post-surgical complications (4, 12, 13). The Apfel scale predicts a PONV incidence of 60–80% in subjects with moderate to severe risks (8). However, our study reported a PONV incidence of 33%; these results support our hypothesis that application of TDS within 2 h prior to surgery and the IV administration of ondansetron and dexamethasone immediately after anesthesia induction is an alternative regimen to reduce the incidence of PONV in subjects undergoing craniotomy under general anesthesia.

The literature describes adverse events associated with the use of TDS as generally mild (1, 2, 21, 27). The most frequent adverse event reported is dryness of mouth (29–91%); and other less common drug reaction are drowsiness (<16.6%), dizziness (8–12%), blurred vision (<8%), and disorientation (<1%) (1, 2, 21, 27). The results from our study did not show significant incidence of any of the most common adverse events related with TDS; particularly dry mouth, which is a symptom that could be difficult to assess in neurosurgical settings and commonly reported preoperatively settings as well (21). In addition, our results showed that the administration of this prophylactic regimen did not significantly increase the QTc interval in the ECGs.

The incidence of PONV in patients with three Apfel’s risk factors is 60% (recorded in the literature) (8) and our results showed an incidence of only 26.7% (8) (Table 4). Gan et al. confirmed the effectiveness of TDS with data collected from 0 to 48 h postoperative; they included one of the largest sample size populations to date (10). The study assessed the effectiveness of the use of TDS plus ondansetron compared with a placebo patch plus ondansetron as a preventing treatment for PONV in approximately 620 female patients that underwent outpatient laparoscopic or breast augmentation procedures (10). This study found a significant reduction in PONV 24 h after surgery in the group who received a combination of TDS and ondansetron; 48% of subjects that received a combination of TDS and ondansetron and 39% that received only ondansetron did not experience vomiting and did not require rescue medication (10). Even TDS has a slow onset of action, this study showed that clinical benefits seem when TDS is applied 2 h before induction of anesthesia when it is used in a combination with ondansetron (10). In addition, this trial reported that the overall incidence of adverse events was less frequent in the group receiving TDS in combination with ondansetron compared with the group receiving ondansetron alone (10).

**Table 4:** **Apfel comparison in PONV 24 h: literature versus our findings**.

Apfel risk factor	*N*	PONV 24 h	PONV 24 h (Apfel) (%)	*p*-Value	CI
2	13 (36.1%)	2 (15.4%)	40	0.071	[0.04, 0.42]
3	15 (41.7%)	4 (26.7%)	60	0.008	[0.11, 0.52]
4	8 (22.2%)	6 (75.0%)	80	0.723	[0.41, 0.92]

Latz et al. presented a prospective study that evaluated the incidence and risks factors of PONV in 229 subjects after craniotomies (5). They found an incidence of PONV during the first 24 h after surgery of 47% and identified absence of intraoperative steroids as a risk factor (5). In our study, the use of dexamethasone as a potent antiemetic on the triple therapy on subjects after craniotomy reduced PONV considerably.

Lee HK et al. demonstrated that the combination of TDS plus dexamethasone was more effective in complete remission of PONV compared with dexamethasone alone or dexamethasone plus ramosetron (82.5 versus 47.5, and 50.0%, respectively) in 120 subjects who underwent major orthopedic surgery with patient-controlled analgesia via epidural route (15). A few limitations of this study included the lack of a control-group without prophylaxis, PONV assessments were performed only for 24 h after surgery and all subjects had an indwelling urinary catheter; thus, the side effect of TDS on urinary retention was not assessed (15, 16).

Habib et al. designed a study comparing the combination of aprepitant and dexamethasone versus ondansetron and dexamethasone for PONV prophylaxis in subjects that underwent craniotomy (4). They reported an accumulative incidence of PONV during the first 24 h, 36% with ondansetron and 14% with aprepitant. However, the difference of nausea and rescue medication was not statistically significant (4).

Lee et al. designed a similar randomized study that assessed the efficacy of the administration of TDS and ondansetron IV to prevent nausea after uterine artery embolization compared with ondansetron IV alone (16). Overall, the incidence of nausea after this procedure was low; there was a lower level of nausea with those treated with TDS compared with placebo during the first 24 h after embolization (16). Adverse events were more common in the TDS group, with two subjects experiencing episodes of profound disorientation and 71% reported considerable dry mouth (16). Their results showed that TDS offers moderate reduction of nausea; but, it was associated with occasional but notable episodes of disorientation. Therefore, according to their findings, the decision of using TDS should be based on careful consideration of the potential benefits and undesirable side effects (16). No adverse events associated with scopolamine were found (16).

The literature reported an incidence of nausea after craniotomy considerably variable, ranging from 43 to 70% (4–7) and vomiting from 33% (28) to 55% (5). In another study designed by Fabling et al., a randomized double blinded study compared ondansetron, droperidol, and placebo after supratentorial craniotomy showed that the incidence of PONV during the first 24 h was 35, 30, and 70%, respectively (6).

Our study had its limitations that should be considered. First, it was a pilot study with the analysis of 36 subjects with no control group. Its primary objective was the assessment of efficacy and safety of triple therapy with TDS in craniotomies. Second, ondansetron was administered during induction of anesthesia as recommended in the package insert rather than used at the end of surgery as suggested by the latest PONV guidelines (1). Third, all subjects left the operation room with an indwelling urinary catheter, and thus the effects of TDS on urinary retention could not be assessed. In addition, TDS was removed 24 h after the end of surgery; future studies should maintain the TDS for 72 h and assess efficacy for PONV and PDNV. In addition, we acknowledge another crucial limitation form this pilot study, which is the difficulty to properly assess common adverse events (dry mouth, drowsiness, dizziness, etc.) associated with TDS application in the neurosurgical setting. Therefore, our results need to be interpreted with caution and new prospective, control-group and randomized trials are needed to address these limitations.

## Conclusion

The primary efficacy of the study was to determine the efficacy and safety of using a prophylactic triple therapy with TDS, ondansetron, and dexamethasone to prevent PONV. Our data showed a PONV incidence of 33% and accumulative incidence of nausea and vomiting of 38% 120 h after surgery. In addition, our results showed that the triple therapy regimen used in this study may be an efficient alternative regimen for PONV prophylaxis in subjects undergoing neurological surgery with general anesthesia. Further studies using regimens affecting different receptor pathways should be performed to better prove the efficacy in preventing PONV.

## Conflict of Interest Statement

The authors declare that the research was conducted in the absence of any commercial or financial relationships that could be construed as a potential conflict of interest.
